# Does Self-Efficacy and Emotional Control Protect Hospital Staff From COVID-19 Anxiety and PTSD Symptoms? Psychological Functioning of Hospital Staff After the Announcement of COVID-19 Coronavirus Pandemic

**DOI:** 10.3389/fpsyg.2020.552583

**Published:** 2020-12-23

**Authors:** Monika Bidzan, Ilona Bidzan-Bluma, Aleksandra Szulman-Wardal, Marcus Stueck, Mariola Bidzan

**Affiliations:** ^1^Medical University of Gdansk, Gdańsk, Poland; ^2^University of Gdansk, Gdańsk, Poland; ^3^The Specialist Hospital in Koscierzyna, Koscierzyna, Poland; ^4^DPFA Academy of Work and Health, Leipzig, Germany

**Keywords:** COVID-19, healthcare workers, disaster, fears, protective factors

## Abstract

**Objectives:**

The aim of this study was to assess coronavirus disease 2019 (COVID-19) anxiety and posttraumatic stress disorder (PTSD) symptoms in the hospital staff, as well as to identify protective factors of COVID-19 anxiety once the coronavirus pandemic was announced in Poland.

**Methods:**

90 healthcare workers from the hospital in Poland completed validated self-report questionnaires assessing self-efficacy, emotional control, and PTSD symptoms; a questionnaire assessing COVID-19 anxiety; and a socio-demographic questionnaire. A multiple linear regression was conducted to assess the effects of gender, being directly vs indirectly exposed to patients, and general self-efficacy on COVID-19 anxiety.

**Results:**

The analysis showed that female (*β* = −0.271, *p* < 0.01) healthcare professionals indirectly exposed to patients (*β* = −0.336, *p* < 0.01) and those who reported lower levels of general self-efficacy (*β* = −0.295, *p* < 0.01) have a stronger tendency to experience COVID-19 anxiety [*R*^2^ = 0.301, *F*(3,89) = 12.34, *p* < 0.01].

**Conclusion:**

The findings show the importance of self-efficacy for dealing with COVID-19 anxiety. The internal coping strategies should be introduced to healthcare workers.

## Introduction

Coronavirus disease 2019 (COVID-19) is thought to be a highly infectious disease. It is primarily transmitted by respiratory droplets and has a similar incubation time and generation time as SARS coronavirus (SARS-CoV) (Wilder-Smith and Freedman, 2020). The first case of COVID-19 had been reported in Wuhan City, China, on 9 January 2020 (Lu et al., 2020). Despite Wuhan City being locked down within 2 weeks after COVID-19 had been reported, the novel virus has soon reached other provinces in China and neighboring countries. By 11 March 2020, the World Health Organization (WHO) declared COVID-19 a global pandemic. At the time we started writing this article (13 April 2020), there were over 1.4 million confirmed cases of COVID-19 and 116,052 deaths globally; at the time we finished it (12 June 2020), there are 7,616,598 confirmed cases of COVID-19 and 424,227 deaths globally (Khasawneh, 2020; Medonet, 2020; Worldometers, 2020). 215 countries are affected by the novel coronavirus (Worldometers, 2020), including Poland, where the first case of COVID-19 had been reported on 4 March 2020, and the COVID-19 pandemic had been announced on 20 March 2020.

It is worth bearing in mind that since the Ebola virus pandemic back in 1976, COVID-19 pandemic has been the one and only pandemic outbreak from among 25 recognized EVD pandemics, which “spread all over the world” and fulfilled the criteria of a “pandemic” (Espinola et al., 2016; Rabelo et al., 2016; Shultz et al., 2016). Widespread outbreak of COVID-19 has frightened and alerted the whole world. This might be partly due to media, which has been constantly updating the global population with the news on COVID-19 outbreak. Fear behaviors in a situation of a disaster spread rapidly and contagiously (Espinola et al., 2016; Shultz et al., 2016), which has resulted in a global panic (Vellingiri et al., 2020). In Poland, the “pandemic fear” had started before the pandemic was announced. For example, “panic buying” (Sim et al., 2020) had started before the lock-down on 11 March 2020.

COVID-19 affects both the physical and mental health of the affected population (North et al., 2004; Khan et al., 2020; Lai et al., 2020; Super et al., 2020). During a disaster, mental disorders are often diagnosed in an affected population, such as adjustment disorders, depression, posttraumatic stress disorder (PTSD), anxiety disorders, non-specific somatic symptoms, and substance abuse (North, 2002; North et al., 2004; Liu et al., 2006; Tsai et al., 2007; Frankenberg et al., 2008; Hollifield et al., 2008; Wu et al., 2009; Math et al., 2015; Bidzan-Bluma et al., 2020; Lai et al., 2020; Khasawneh, 2020; Rajkumar, 2020; Super et al., 2020).

Researchers have assigned PTSD as the signature diagnosis among post-disaster mental morbidity (North, 2003; Math et al., 2015; Ogińska-Bulik and Kraska, 2017; Moghadam et al., 2020). The main PTSD symptoms are intrusion (reliving the traumatic event over and over again in dreams or memories), avoidance (of feelings, conversations, stimuli, or actions related to the event experienced), and agitation (trouble with focusing, but also falling asleep, general irritability, strong emotional reactions in response to sudden stimuli, constant sense of threat) (World Health Organization [WHO], 1992; Juczyński and Oginìska-Bulik, 2009; Juczyński, 2012; Rybojad and Aftyka, 2018). According to DSM-5 (2013), diagnosis of PTSD includes experiencing repeated or extreme exposure to aversive details of the traumatic events, occurring usually during performance of professional duties. In the case of disasters, this mainly refers to the emergency services (police, military, fire fighters, paramedics) (James, 2011a,b; Ogińska–Bulik, 2013, 2016; Ogińska-Bulik and Juczyński, 2016; Rybojad and Aftyka, 2018). In relation to COVID-19, this applies primarily to the medical staff providing direct help to the infected patients and those under the threat of developing the disease. However, the incidence of PTSD in these groups is highly diversified and depends on factors such as the scope of exposure, social support, and training (James, 2011a,b; Ogińska–Bulik, 2013; Ogińska-Bulik and Juczyński, 2016). In particular, demographic characteristics such as age and gender are associated with different rates of PTSD, with younger people and women more likely to develop this stress disorder. Interpersonal and psychological characteristics of the individual, such as social support and self-esteem, have also been implicated in the onset and course of PTSD (Adams and Boscarino, 2006).

The medical personnel, including paramedics, physicians, nurses, obstetricians, nursing aids, psychologists, but also medical analysts, radiology professionals, and cleaning, transport, and technical staff, are all involved in a particular way in preventing the consequences of COVID-19 pandemic and performing crisis interventions. Healthcare workers should be regarded as a highly exposed group with a higher risk of psychiatric symptoms during the COVID-19 pandemic. The risk factors among healthcare workers include female gender and being a frontline worker among others (Vindegaard and Benros, 2020).

Among Chinese healthcare workers exposed to COVID-19 (from clinics or wards for patients with COVID-19 in multiple regions of China), women, nurses, individuals living in Wuhan, and frontline healthcare workers have been shown to have a high risk of developing unfavorable mental health outcomes. They reported experiencing symptoms of depression, anxiety, insomnia, and distress, especially female nurses (Lai et al., 2020).

Personal and social resources are constituting means of efficient coping with a threat. Studies have confirmed a positive meaning of the possibility to express emotions when faced with a disaster. Personal resources of an individual play a crucial role in emotional expression. These include the sense of personal control, self-efficacy, resourcefulness, sense of humor, optimism, valuation, and coping with distressing events (Adams and Boscarino, 2006; Doliński, 2006; Ranieri et al., 2020). Medical profession representatives are expected to have psychological resilience, whose significant aspect is experiencing emotions and expressing them in a certain way. Experiencing emotions is usually accompanied by somatic changes, mimic and pantomimic expressions, and specific behavior (Da̧browska-Chołostiakow and Kocbach, 2018). Emotions are related to self-control, which is defined as demonstrating a behavior consistent with the norms accepted by an individual or with social norms. Self-control involves reactions initiated by a subject, by means of which he achieves congruence among his own emotional states, thought and affective feelings, and the accepted internal norms (internalized rules of functioning) or the external ones (socially approved rules) (Wagner and Heatherton, 2013). It is emphasized in the literature that suppressing emotions is an unfavorable phenomenon, because it may lead to intensification of the experienced emotions or their lingering in the form of emotional tension (Doliński, 2006).

Self-efficacy allows to assess the situations accurately and seek efficient ways of coping with the encountered difficulties and obstacles (Juczyński, 2000, 2012). People with high self-efficacy can maintain relatively stable emotions even under pressure (Bihlmaier and Schlarb, 2016). Self-efficacy also increases concentration and self-control (Przepiórka et al., 2019; Xiao et al., 2020). Low sense of self-efficacy is related to anxiety and sense of helplessness, while high sense of self-efficacy is related to the higher level of positive emotions (these persons assess distressing stimuli more often as a challenge than a threat), which favors taking up challenges, defining aims, and achieving successes in fulfilling them (Maddux and Lewis, 1995; Juczyński, 2012; Schwarzer, 2015).

Currently, there are no studies conducted on the psychological resources, including emotional control and self-efficacy, of healthcare workers during the COVID-19 pandemic. At the time of the COVID-19 pandemic, the workplace environment is not supportive in Polish hospitals because Polish healthcare centers suffer from acute lack of the necessary protective measures, including protective masks, disposable gloves, protective coats and coveralls, and disinfecting liquids. This makes healthcare workers particularly vulnerable to developing unfavorable mental health outcomes, including PTSD. Based on the previous studies, it seems that the sense of self-efficacy and emotional control (of anger, anxiety, depression) may be related to the functioning of the hospital staff, including the intensity of PTSD, after the COVID-19 pandemic was announced.

The aim of this study was to assess the psychological resources, including coping self-efficacy and emotional control (of anger, anxiety, depression) of hospital staff with reference to the coronavirus anxiety and PTSD symptoms after the coronavirus pandemic was announced.

This is the first study to investigate the psychological resources of healthcare workers during the COVID-19 pandemic, and the first study to assess psychological outcomes of the COVID-19 pandemic on healthcare workers in Europe.

## Materials and Methods

### Study Group

The study group consisted of 90 healthcare workers from a district hospital in Kościerzyna, Pomeranian region, Poland. The staff included paramedics, physicians, obstetricians, nursing aids, psychologists, medical analysts, radiology specialists, cleaning, transport, and technical crews. Descriptive characteristics of the study participants are described in Table 1.

**TABLE 1:** Descriptive characteristics of the study participants.

Variables	*N* = 90
Gender	
Males	*N* = 23 (25.6%)
Females	*N* = 67 (74.4%)
Direct vs indirect exposure to patient	
Direct	*N* = 66 (73.3%)
Indirect	*N* = 24 (26.7%)
Specialization	
Yes	*N* = 48 (53.3%)
No	*N* = 42 (46.7%)
Age	*M* = 45.66
	(*SD* = 9.70)
Number of years in the profession	*M* = 19.373
	(*SD* = 11.29)

### Research Procedure

The study was conducted in the hospital on 22 March 2020—2 days after the coronavirus pandemic had been announced in Poland. Considering that the lockdown-type control measures in Poland started before the pandemic was announced (first lockdown-type control measures had started on 10 March 2020), the panic had also started well before the pandemic was announced.

The research project was reviewed and approved positively by the Ethical Committee (decision no. 29/2020) at the Institute of Psychology at the University of Gdańsk, Poland. Participants in the current study were obtained with a cooperation of a gatekeeper, which allowed access to the hospital. Participants included in the study were hospital workers, without any psychiatric diagnosis in the past to the present, who agreed to participate in the study. Exclusion criteria included DSM-5 psychiatric diagnoses (American Psychiatric Association, 2013) and taking psychotropic medications. Psychiatric diagnoses were performed by a psychiatrist.

Recruitment of the participants involved a general conversation about the pandemic, which was meant to encourage the respondent to take part in the study. After agreeing to take part in the study, individual meetings were scheduled. During the meetings, participants were asked to fill out the questionnaires.

The following research tools were used:

1. Author’s own survey including socio-demographic data
2. Author’s own three-item anxiety scale concerning anxiety about coronavirus.

The respondents were asked to assess, on a scale of 0–10, how strong are their fears of COVID-19 in relation to: worries about themselves (I am worried about myself), worries about loved ones (I am worried about my loved ones), and fear of losing their job (I am worried about losing my job and poverty). Responses for all three items were summed up to create a composite measure in which higher scores reflect greater anxiety concerning the consequences of COVID-19. This is an author-designed tool, which has been standardized in relation to Hamilton Anxiety Rating Scale (HAM-A). Scores of 15 and above correspond with HAM-A scores of 18 and above, which is a commonly used cutoff score of anxiety disorders in clinical studies (Hamilton, 1959; Małyszczak et al., 1999; Kocjan, 2016).

A one-factor structure of the tool was tested via confirmatory factor analysis (CFA). Model’s fit statistics show very good fit: CFI = 0.99, RMSEA = 0.001. Scale reliability in the current study was assessed using the Cronbach’s alpha. The results of this measure equaled 0.77.

In addition, the measurement of COVID-19 anxiety was supplemented with a single item assessing concerns about coronavirus infection, i.e., “Are you worried about catching the coronavirus?” Study participants reported their concerns on a five-point scale: (1) Definitely not; (2) Probably not; (3) Probably yes; (4) Yes; and (5) Definitely yes. On the scale, scores of 3 and above indicated fear of catching COVID-19; hence, we selected 3 as a cutoff score.

Considering a relatively small number of participants (*N* = 90), we used a dichotomic classification of fear of COVID-19 for both scales. For the dichotomic classification, we used the cutoff scores and assigned 0 = no fear, 1 = fear. This could result less likely to creation of biased estimates than are more unbalanced dichotomies (Pedhazur and Schmelkin, 1991; Borz and Döhring, 2006).

General Self-Efficacy Scale (GSES), which is a 10-item psychometric scale that is designed to assess optimistic self-beliefs to cope with a variety of difficult demands in life. The scale was originally developed in German by Jerusalem and Schwarzer (1992). The total score a person can get is between 10 and 40 points. The higher the score, the higher the self-efficacy, which translates into greater self-confidence and better ability to cope with a difficult situation. Sten scores 10–24 points were regarded as low, 30–40 points as high, and 25–29 points as average.

A Polish version (Juczyński, 2012) has been shown to be reliable and valid (Cronbach’s alpha equaled 0.85). Additionally, scale reliability was assessed by the author using the test–retest method (after 5 weeks) and equaled 0.78 (Juczyński, 2012); in the present study, Cronbach’s alpha resulted to be 0.86.

The Courtauld Emotional Control Scale (CECS) (Polish version), which is a 22-item questionnaire developed to measure the extent to which individuals report suppressing emotions of anger, anxiety, and depressed mood. Subscales have been shown to be consistent with these primary emotions: anger, depressed mood, and anxiety. The total emotional control index is within 21–84 points. The higher the result, the more enhanced the suppression of negative emotions. The Polish version was found to be a reliable and valid method. The following Cronbach’s α coefficients were obtained: for the control of anger 0.80, depression 0.77, anxiety 0.78, and for the total emotional control index 0.87 (Juczyński, 2012), with our data Cronbach’s alpha for the total emotional control index equaled 0.76.

Impact Event Scale-Revised (IES-R)—Polish version (Juczyński and Oginìska-Bulik, 2009) is a self-report measure that assesses a subjective psychological distress caused by a traumatic event. The principal component analysis identified three factors (Intrusion, Hyperarousal, and Avoidance), which are closely associated with PTSD symptoms. The cutoff point is 30 points.

The Polish version was found to be a reliable and valid method. The overall scale reliability measured with Cronbach’s alpha is 0.92; in the present study, Cronbach’s alpha resulted to be 0.94.

### Data Analyses

Descriptive statistics were computed for the characteristics of the sample consisting of frequencies and percentages for categorical variables and means and standard deviations (SDs) for scale variables (Table 1). Parametric assumptions were tested before conducting parametric tests. Differences in levels of anxiety concerning the consequences of COVID-19 between males and females; professionals directly vs indirectly exposed to patients; and professionals with vs without specialization were assessed via *t*-test.

Before analyzing the correlation between variables, we assessed the normality of the distribution of variables on the basis of skewness and kurtosis (see Table 2).

**TABLE 2:** Means, standard deviations, and correlation matrix of the study variables.

Variables	*M*	*SD*	Skew.	Kurt.	1	2	3	4	5	6
1. Age	45.66	9.70	–0.51	–0.32						
2. Number of years in the profession	19.37	11.29	0.05	–0.85	0.790**					
3. Fear of catching COVID-19	3.97	1.09	–0.63	–0.93	0.031	0.061				
4. Anxiety concerning the consequences of COVID-19	19.49	8.10	–0.20	–1.06	0.114	–0.040	0.478**			
5. GSES	30.66	3.82	0.66	1.45	–0.040	–0.090	−0.331**	−0.322**		
6. CECS overall	49.74	9.31	0.37	–0.03	−0.304**	–0.160	–0.040	0.030	–0.160	
7. PTSD overall (IES-R)	62.16	18.21	0.01	–0.70	0.239*	0.209	0.488**	0.348**	−0.253*	−0.140

*N = 90.^∗^p < 0.05.^∗∗^p < 0.01.*

The criterion set was < 2.0.

As there were no clear deviations from the normal distribution, we used the Pearson’s *r* coefficient. Bivariate associations between study variables were assessed via Pearson’s correlation coefficient *r* (Table 2).

A multiple linear regression was conducted to ascertain the effects of gender, being directly vs indirectly exposed to patients, and general self-efficacy on the anxiety concerning the consequences of COVID-19. We followed the hypothesis, and CECS is not the significant predictor, so we did not include it in the regression model.

## Results

The majority of the respondents (*N* = 62; 68.9%) reported being worried of catching COVID-19. The majority of the respondents (*N* = 84, 93%) also demonstrated a subjective distress caused by COVID-19 (only six individuals did not report them). The mean General Self-Efficacy GSES was relatively high in the studied group of respondents. The mean score of Control of Negative Emotion CECS suggests that the respondents had an average suppression of negative emotions. Table 2 contains descriptive statistics and a correlation matrix (Pearson’s correlation) of the variables studied.

Further analysis showed that women reported higher levels of anxiety concerning the consequences of COVID-19 (*M* = 20.82, *SD* = 7.53) than men (*M* = 15.61, *SD* = 8.23), *t*(88) = 2.76, *p* < 0.01; the difference is moderate (Cohen’s *d* = 0.66) (equal variance assumed *F* = 0.24, *p* = 0.63). Contrary to expectations, healthcare professionals directly exposed to patients reported lower levels of anxiety concerning the consequences of COVID-19 (*M* = 17.40, *SD* = 7.92) than those who were indirectly exposed to patients (*M* = 24.11, *SD* = 6.53), *t*(88) = 3.92, *p* < 0.0; the difference was high (Cohen’s *d* = 0.92) (equal variance assumed *F* = 1.20, *p* = 0.23). Healthcare workers with specialization obtained significantly lower mean scores for anxiety concerning the consequences of COVID-19 (*M* = 16.27, *SD* = 7.32) than workers without specialization (*M* = 22.57, *SD* = 7.67), *t* = 3.98, *p* < 0.01; the difference was high (Cohen’s *d* = 0.84) (equal variance assumed *F* = 0.31, *p* = 0.58). There were no significant differences between healthcare workers with and without specialization when PTSD symptoms overall score was compared (*t* = 1.56, *p* = 0.12).

### Linear Regression Analysis

A multiple linear regression was conducted with SPSS Statistics to ascertain the effects of gender, being directly vs indirectly exposed to patients, and general self-efficacy on the anxiety concerning the consequences of COVID-19. The results of multiple regression analyses showed a significant main effect of all predictors on the anxiety concerning the consequences of COVID-19, suggesting that female (*β* = −0.271, *p* < 0.01) healthcare professionals indirectly exposed to patients (*β* = −0.336, *p* < 0.01), who reported lower levels general self-efficacy (*β* = −0.295, *p* < 0.01) have a stronger tendency to respond with anxiety regarding the consequences of COVID-19 [*R*^2^ = 0.301, *F*(3,89) = 12.34, *p* < 0.01].

## Discussion

The aim of this study was to assess the psychological resources, including coping self-efficacy and emotional control (of anger, anxiety, depression) of hospital staff with reference to the coronavirus anxiety and PTSD symptoms, once the coronavirus pandemic was announced in Poland. The study was conducted shortly after the restriction period in Poland has started, and it investigated the subjective assessment of COVID-19 threat and PTSD symptoms in the hospital staff, as well as identified protective factors of fear of COVID-19.

The COVID-19 pandemic was perceived by the group of hospital personnel (medical, technical, and maintenance staff) as a genuine threat. Majority of the respondents were worried of catching COVID-19. According to the stress-adaptation model, the experience of fear and stress is defined as a universally experienced response to extraordinary life circumstances (Maudner et al., 2003; Valdez and Nichols, 2013). The fear of catching the coronavirus may also stem from the high awareness of the hospital staff that the virus may be absorbed by the cells of the mucus membrane in the eyes, nose, cheek—after which it changes its genetic code, multiplies, and transforms its own cells into the cells of the intruder. The virus is invisible and may be everywhere—in a patient’s breath, on his clothes, items in his possession, and on everything he touched. It is easily transmittable. The hospital personnel might thus perceive the virus as a genuine invisible threat to them and all persons they get in touch with (including their close friends and relatives). This could explain their real fear of coronavirus. The perception of one’s own situation as threatening may deepen insecurity. Usually, at the time of pandemic, infection monitoring procedures and public health recommendations are modified frequently. The changes may be introduced on a daily or even hourly basis, which explains the increased level of insecurity in the medical personnel. It seems that media broadcast also intensifies insecurity and anxiety. Research also suggests that the staff lacks both planning and strategic solutions for the community at various levels at the time of disasters, which also intensifies insecurity and anxiety when facing a threat (Roudini et al., 2017). Moreover, when faced with a disaster, individual fear behaviors spread rapidly and contagiously, among groups of persons who share the fear and observe the behaviors of each other (Espinola et al., 2016; Shultz et al., 2016). Finally, the fear of COVID-19 may also be related with stigmatization of healthcare workers, others’ fear of contact with those treating patients with COVID-19 (Ramaci et al., 2020). We could observe similar fear-related behaviors in the years 2013–2016 during the West Africa Ebola Virus Disease Outbreak (Espinola et al., 2016; Rabelo et al., 2016; Shultz et al., 2016).

Widespread outbreak of COVID-19 is associated with psychological distress and symptoms of mental illness (Bao et al., 2020; Rajkumar, 2020; Super et al., 2020; Vindegaard and Benros, 2020). Work-related mental health impairment is recognized as a real problem in the context of helping responders, including health professionals, due to adverse health outcomes after a severe disaster (Neria et al., 2011; Farooqui et al., 2017; Nukui et al., 2018). Recent studies have shown that among healthcare workers depression and anxiety rates were higher (Chen et al., 2020; Mo et al., 2020) compared to administrative staff (Lu et al., 2020; Zhang et al., 2020) and non-frontline workers (Liang et al., 2020; Vindegaard and Benros, 2020), during the COVID-19 pandemic (Xu et al., 2020). We observed differences between workers directly exposed to patients and those indirectly exposed to patients. Despite the fact that workers directly exposed to patients are more prone to being directly exposed to COVID-19, they experienced coronavirus-related worries less frequently than workers indirectly exposed to patients, as well as healthcare workers with specialization obtained significantly lower mean scores for anxiety concerning the consequences of COVID-19 than workers without specialization. This could be explained by their awareness of having a job which is strongly socially desirable, as well as awareness of their own skills and the sense of self-efficacy. Some research has shown contradicting results, i.e., female nurses in Wuhan working in the front-line medical staff were twice more likely to suffer anxiety and depression than the non-clinical staff (Lai et al., 2020; Lu et al., 2020). In those studies, however, studied nurses have had close contact with COVID-19 patients. In the present study, the medical staff could potentially have had a direct contact with patients infected with COVID-19, yet there were no cases of COVID-19 registered in the hospital.

In the current study, gender was another variable that turned out to be a significant predictor to respond with anxiety regarding the consequences of COVID-19. Some studies have revealed that female gender and younger age are some of the risk factors of anxiety (Norris et al., 2002; Frankenberg et al., 2008; Math et al., 2015; Sohrabizadeh et al., 2016; Vindegaard and Benros, 2020). Younger persons and women are also more likely to develop that stress disorder when faced with a disaster (Adams and Boscarino, 2006). Similarly, disaster rescue workers are at high risk of developing psychiatric morbidity (Stellman et al., 2008).

In the current study, the vast majority of the participants (apart from six respondents) declared PTSD symptoms. A mass threat is a potentially traumatic event (PTE) that threatens or overtly endangers the physical and/or psychological health, well-being, and integrity of a population and that is perceived and experienced, both individually and collectively, by persons comprising the population at risk. PTEs, regardless of whether they result in physical harm, have the capacity to produce psychological distress and, with severe or prolonged exposure, PTSD (Espinola et al., 2016). It is worth emphasizing that not all respondents declared being anxious of COVID-19, and not all of them had the PTSD symptoms. Although researchers have assigned the PTSD as the signature diagnosis among post-disaster mental morbidity, the incidence of PTSD reported in literature ranges from 4 to 60% (Pietrzak et al., 2012; Brooks et al., 2019). The level of PTSD symptoms was correlated with age, sense of self-efficacy, and fear of catching COVID-19 and anxiety concerning the consequences of COVID-19. There was no significant relationship between gender and PTSD symptoms scores.

Nowadays, the workplace environment is not supportive in Polish hospitals, especially at the time of COVID-19 pandemic, because currently Polish healthcare centers suffer from acute lack of the necessary protective measures, including protective masks, disposable gloves, protective coats and coveralls, and disinfecting liquids. We assumed that emotional control would be a significant factor protecting against the PTSD symptoms and fear of COVID-19. The obtained results do not confirm this assumption. Only age negatively correlated with emotional control. Younger age supported the control of negative emotions, which could be due to greater adherence to the professional workplace norms, which favor emotional control and discourage expressing negative emotions (anger, anxiety, depression) when at work in a hospital. The control of negative emotions decreased with age. The obtained results are consistent with Averill’s (2004) review. According to Averill (2004), the diversity of emotions experienced by a person results from the possession of various cognitive emotional programs of responding to events. Cognitive emotional patterns are inborn, but they develop and change under the impact of life experiences. Emotions are a kind of social role. Controlling them means being emotionally correct, which is a condition of high emotional intelligence (Averill, 2004). The fact that the hospital staff do not always control negative emotions or admit much more easily to it may be due to the general life experience growing with age. Expressing negative emotions is beneficial and recommended in various psychotherapeutic approaches (Salovey et al., 2002).

We assumed that the sense of self-efficacy will be a significant resource in the light of the COVID-19 pandemic. The GSES scores were negatively correlated with fear of catching the coronavirus as well as anxiety concerning the consequences of COVID-19. The sense of self-efficacy of hospital personnel is based primarily on their education, practical skills, and expert knowledge, so even lack of external resources, such as protective masks they have, face shields, goggles, disposable gloves, protective coats and coveralls, and disinfecting liquids, does not lower the sense of self-efficacy in all hospital staff groups. Anxiety has been shown to increase sensitivity to work pressure and the working environment and has a negative effect on self-efficacy because it reduces positive behaviors and initiative (Bandura and Adams, 1977; Miller et al., 2006; Xiao et al., 2020).

### Strengths

The main strength of the study is that it was conducted 2 days after the COVID-19 pandemic had been announced in Poland, which controls the limitations that often arise in retrospective studies. Furthermore, it was conducted in a non-artificial environment—at the workplace, with direct researcher–respondent contact. The study group consisted of the staff from one hospital, who were directly, as well as indirectly, exposed to patients. Personnel not commonly involved in research, such as maintenance workers, were also included in this study.

### Limitations

The limitations of this study include a sample that is limited to the personnel in only one hospital in the Pomeranian region, Poland, which makes it impossible to generalize the conclusions to hospital staff from other hospitals. Moreover, the current study investigated symptoms and/or signs of PTSD rather than PTSD. It is hence impossible to ascertain whether the respondents suffered from PTSD or only experienced the symptoms of PTSD. In order to diagnose PTSD, a full psychiatric assessment would need to be conducted.

Conducting the study just 2 days after the COVID-19 pandemic had been announced in Poland can be a strength as well as a limitation. Considering that the media broadcast has been intensifying insecurity and anxiety since the first case of COVID-19 had been reported in Wuhan City, it seems that the “coronavirus fear” in Poland had started before the pandemic was announced, yet this is only an assumption. Symptoms of PTSD usually manifest within the first 3 months after the trauma, hence not enough time might have passed for some of the respondents to manifest PTSD symptoms. It is also difficult to ascertain without a psychiatric examination whether the respondents had PTSD symptoms or acute stress disorder (ASD) symptoms. PTSD refers to the long-term aftermath of trauma (when the symptoms last longer than a month), while ASD refers to the initial traumatic symptoms that arise immediately after a traumatic event (Bryant et al., 2000a,b; Bryant and Harvey, 2002). PTSD can follow ASD, but it can also occur even when ASD does not develop.

## Conclusion

The findings show the importance of self-efficacy for dealing with COVID-19 anxiety. The internal coping strategies should be introduced to healthcare workers as a part of the psychological preparation and health management to increase the psychological resilience of the hospital staff. Research has shown (Stueck, 2007, 2009; Stueck and Villegas, 2008; Stueck et al., 2019; Parker et al., 2020) that stress reduction methods combined with body orientation, e.g., breathing meditation and Autogenic Training, has a positive effect on self-efficacy. It seems that introducing a modern biocentric and psychological disaster management approach into hospitals could prepare the hospital staff to better deal with a pandemic or crisis.

## Data Availability Statement

The raw data supporting the conclusions of this article will be made available by the authors, without undue reservation.

## Ethics Statement

The studies involving human participants were reviewed and approved by the Ethics Board for Research Projects at the Institute of Psychology, University of Gdansk, Poland. The patients/participants provided their written informed consent to participate in this study.

## Author Contributions

MoB and IB-B contributed to conceptualization, methodology, formal analysis, writing, and original draft preparation. AS-W contributed to conceptualization, investigation, and project administration. MS contributed to conceptualization, writing, and original draft preparation. MaB contributed to conceptualization, methodology, formal analysis, writing, original draft preparation, and supervision. All authors contributed to manuscript revision, read, and approved the submitted version.

## Conflict of Interest

The authors declare that the research was conducted in the absence of any commercial or financial relationships that could be construed as a potential conflict of interest.
